# Joinpoint regression and age-period-cohort analyses of global and Chinese acute hepatitis C incidence trends, 1990–2021

**DOI:** 10.3389/fpubh.2026.1634028

**Published:** 2026-04-02

**Authors:** Menghan Wu, Cheng Hong, Yanjiao Fang, Jin Tao, Xieyu Zhang, Guoming Cheng, Min Zhang, Xianqing Tao, Yuying Li, Mengya Ding, Zhengdong Dou

**Affiliations:** 1 School of Public Health, Wannan Medical College, Wuhu, Anhui, China; 2 Wuhu Center for Disease Control and Prevention, Wuhu, Anhui, China

**Keywords:** acute hepatitis C, age-period-cohort, global burden of disease, incidence, Joinpoint regression

## Abstract

**Objective:**

This study aimed to analyse the temporal trends of acute hepatitis C incidence and the independent effects of age, time period, and birth cohort (age–period–cohort effects) from 1990 to 2021 globally and in China.

**Methods:**

Data were obtained from the Global Burden of Disease Study 2021. Joinpoint regression was used to analyse the trends in age-standardized incidence rates (ASIRs). The age–period–cohort model was employed to disentangle the underlying effects of population ageing, temporal changes, and birth cohort risks.

**Results:**

From 1990 to 2021, the global ASIR of acute hepatitis C exhibited an overall declining trend (average annual percent change [AAPC] = −0.38%). However, this trend reversed after 2015, indicating a concerning resurgence. Disparities were pronounced across socio-demographic index (SDI) levels: Low-SDI regions faced the highest burden, while the ASIR in China declined most rapidly (AAPC = −1.55%). The age–period–cohort analysis revealed a bimodal age pattern, with peaks in early childhood (0–4 years) and old age (≥95 years), indicating distinct transmission routes and historical cohort risks.

**Conclusion:**

Despite a long-term decline, the global increase in acute hepatitis C incidence underscores an ongoing public health challenge. Our findings highlight the urgent need to strengthen prevention and screening strategies, particularly in low-SDI regions, among vulnerable age groups, and for high-risk populations in order to achieve the goal of hepatitis C elimination.

## 1 Introduction

Hepatitis C virus (HCV) is a hepatotropic RNA virus that causes both acute and chronic hepatitis, with approximately 58 million people worldwide infected with HCV and 1.5 million new infections occurring each year. HCV infection remains an ongoing public health threat (1). Acute hepatitis C (AHC) is defined as the 6-month period following infection with HCV, a critical phase during which 15–45% of immunocompetent individuals achieve spontaneous viral clearance, while the majority develop chronic infection leading to cirrhosis and hepatocellular carcinoma (2). Although an increasing incidence of acute hepatitis C has been reported in certain high-risk populations, this trend varies considerably across geographic regions. In some high-income countries, rising incidences have primarily been driven by people who inject drugs, whereas in other regions, healthcare-related or vertical transmission remains more prominent (3). This underscores the necessity of expanding screening and prevention efforts beyond clinical settings to actively reach these key populations. Accordingly, hepatitis C screening and prevention strategies differ substantially across regions and countries, reflecting local epidemiological characteristics and healthcare systems. The advent of therapies with direct antiviral agents (DAAs) has revolutionized the treatment of HCV, achieving sustained virological response (SVR) rates of >95% in patients with chronic infections (4). Therefore, optimising strategies for the early identification and treatment of acute HCV is essential to achieve the World Health Organization’s (WHO) goal of eliminating viral hepatitis by 2030. The Global Burden of Disease Study 2021 (5) has significantly contributed to outlining the epidemiological landscape of HCV through systematic data integration. However, the trends in the disease burden of AHC and its underlying causes remain underexplored. Specifically, the lack of studies that analyse the heterogeneity in AHC incidence across regions with different SDI from an APC perspective impedes a comprehensive understanding of how health system performance, historical exposure events, and birth cohort risks collectively shape the epidemic dynamics of AHC.

In this study, we used data from the GBD 2021 database to analyse the trends and temporal distribution of the burden of hepatitis C globally, in China, and in countries with five SDI levels from 1990 to 2021. The aim is to propose precise screening and prevention efforts and provide a comprehensive perspective for developing targeted hepatitis C prevention strategies that address the specific needs of different countries.

## 2 Data and methodology

### 2.1 Data source and variable extraction

The data were obtained from the Global Burden of Disease Study 2021 (5), which uses the International Classification of Diseases (ICD-10) to categorize the diseases studied. The GBD 2021 and Injury Analysis used 100,983 data sources to estimate the burden of 371 diseases and injuries. Counts and age-standardized rates were calculated from 1990 to 2021 for seven global super-regions, 21 regions, 204 countries and territories (including 21 countries with subnational locations), and 811 subnational locations (5). We extracted the following variables for the disease “Acute hepatitis C”: number of incident cases, deaths, disability-adjusted life years (DALYs), years of life lost (YLLs), and years lived with disability (YLDs). Data were extracted for the ‘Global’ region, ‘China’, and for countries categorized under the five Socio-demographic Index (SDI) groups: low, low middle, middle, high middle, and high SDI. The time span of the study was from 1990 to 2021. The data were disaggregated by sex (both male and female individuals) and by 5-year age groups from 0–4 to 95 + years (20 groups in total).

### 2.2 Data standardization and adjustment for under-reporting

All extracted rates (incidence, death, DALYs, YLLs, and YLDs) are presented as age-standardized rates (ASRs) per 100,000 population. The GBD 2021 uses a standardization method based on the age structure of the GBD world population to ensure comparability across time and geography. The GBD estimation process employs complex statistical models (e.g., spatiotemporal Gaussian process regression and Cause of Death Ensemble modelling) to adjust for under-reporting, missing data, and variations in diagnostic and reporting quality across countries and over time. However, these modelling adjustments and inherent smoothing may reduce sensitivity to very short-term or highly localised fluctuations in disease incidence. The estimates used in this study are the final model-adjusted values from the GBD 2021, and all reported rates include the 95% uncertainty intervals (UIs) provided by the GBD, which reflect the statistical uncertainty of the estimates.

### 2.3 Statistical analyses

#### 2.3.1 Temporal trend analysis

The Joinpoint model was used to analyse the long-term trends in the age-standardized incidence rates (ASIRs) of acute hepatitis C globally, in China, and across SDI groups between 1990 and 2021. The Joinpoint regression model can be expressed as follows: y is the incidence rate, x refers to the year, *β* is a constant term, and *δ*, *τ*, and k denote the regression coefficient, unknown turning point, and number of turning points for each segmented function, respectively (6). In our analysis, the maximum number of Joinpoints was set to 5, and the model selection was based on the permutation test (using 4,499 permutations) and the Bayesian Information Criterion (BIC). The Annual Percent Change (APC) and Average Annual Percent Change (AAPC) were calculated along with their 95% confidence intervals (CIs). An APC/AAPC is considered statistically significant if its 95% CI does not include zero. A positive APC/AAPC ratio indicates an upward trend, whereas a negative value indicates a downward trend in the incidence rate over the specified period.

#### 2.3.2 Age-period-cohort analysis

The APC model was used to analyse the effects of age, period, and birth cohort on the incidence of acute hepatitis C. Age and period were spaced at 5-year intervals, and the cohort was defined as (period-age). The model operates under the key assumption that the effects of age, period, and cohort are additive on a logarithmic scale of the incidence rate. A central challenge in APC analysis is the identifiability problem arising from exact linear dependency (age–period–cohort). To address this, we employed the intrinsic estimator (IE) method, which provides a unique solution by imposing a constraint orthogonal to the null space of the design matrix. This method is widely used in epidemiological studies to obtain stable estimates of age, period, and cohort effects, which are not dependent on arbitrary classical constraints. The effect coefficients of age, period, and cohort were estimated using the IE method, and the relative risk (RR) was obtained.

#### 2.3.3 Statistical processing

In this study, we used Microsoft Excel for data organisation, Joinpoint Regression Program (5.0.0), Joinpoint for time trend analysis, and Stata (17.0) software to construct the APC model, with a test level of *α* = 0. 05. Graphical representations were created using R software (4.4.2).

## 3 Results

### 3.1 Secular trends in ASIR: overall decline with recent resurgence and SDI disparities

From 1990 to 2021, low-SDI countries had the highest standardized incidence of acute hepatitis C, far exceeding the global incidence, and low-middle SDI countries had the second-highest incidence after low SDI; high-SDI countries had the lowest incidence. The global standardized incidence of acute hepatitis C is generally declining, from 103.71 per 100,000 in 1990 to 92.64 per 100,000 in 2021, with an average annual decrease of 0.38% average annual percent change (AAPC = −0.38%, *p* < 0.001). The incidence of acute hepatitis C declined more rapidly in women, from 104.40 per 100,000 in 1990 to 91.69 per 100,000 in 2021, an average annual decrease of 0.44% (AAPC = − 0.44%, *p* < 0.001).

**Figure 1:**
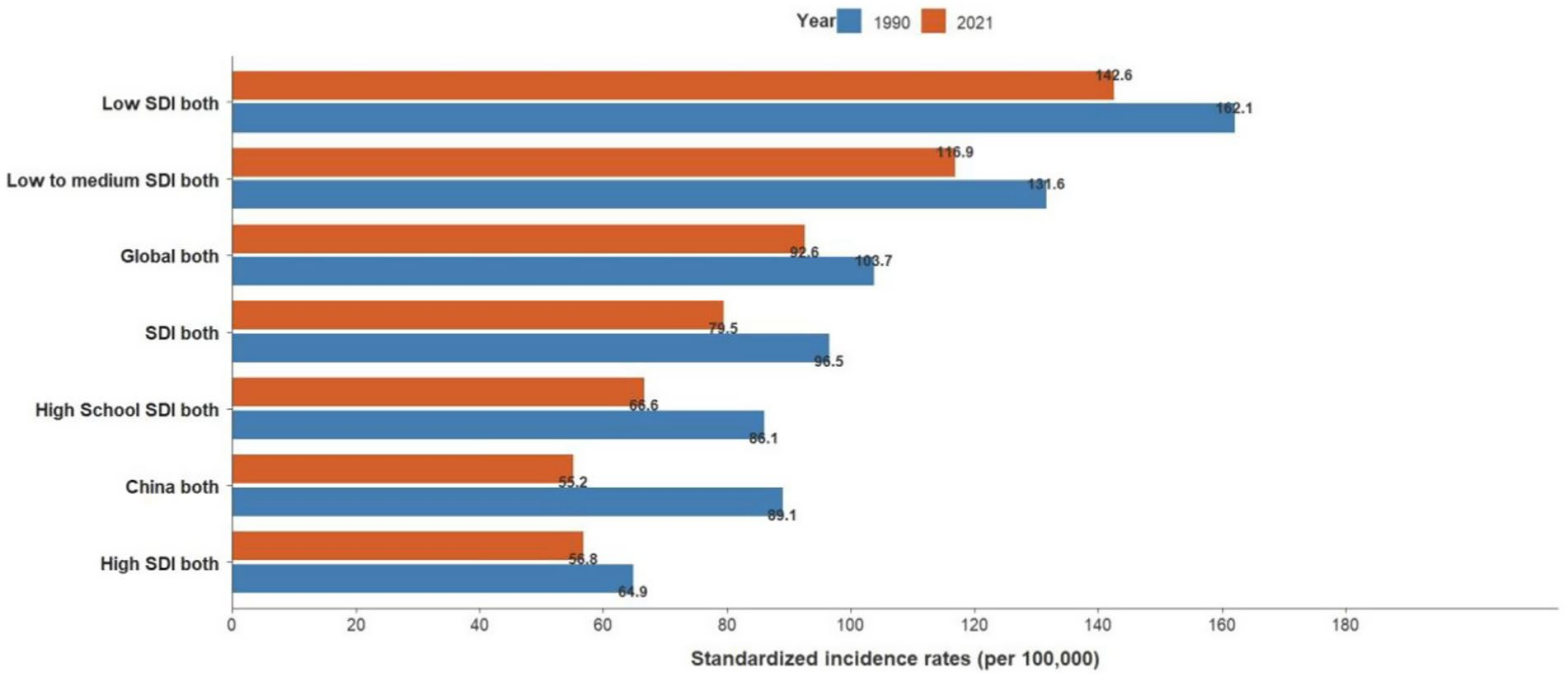
Trends in standardized incidence rates of acute hepatitis C by SDI group, 1990–2021.

**Figure 2:**
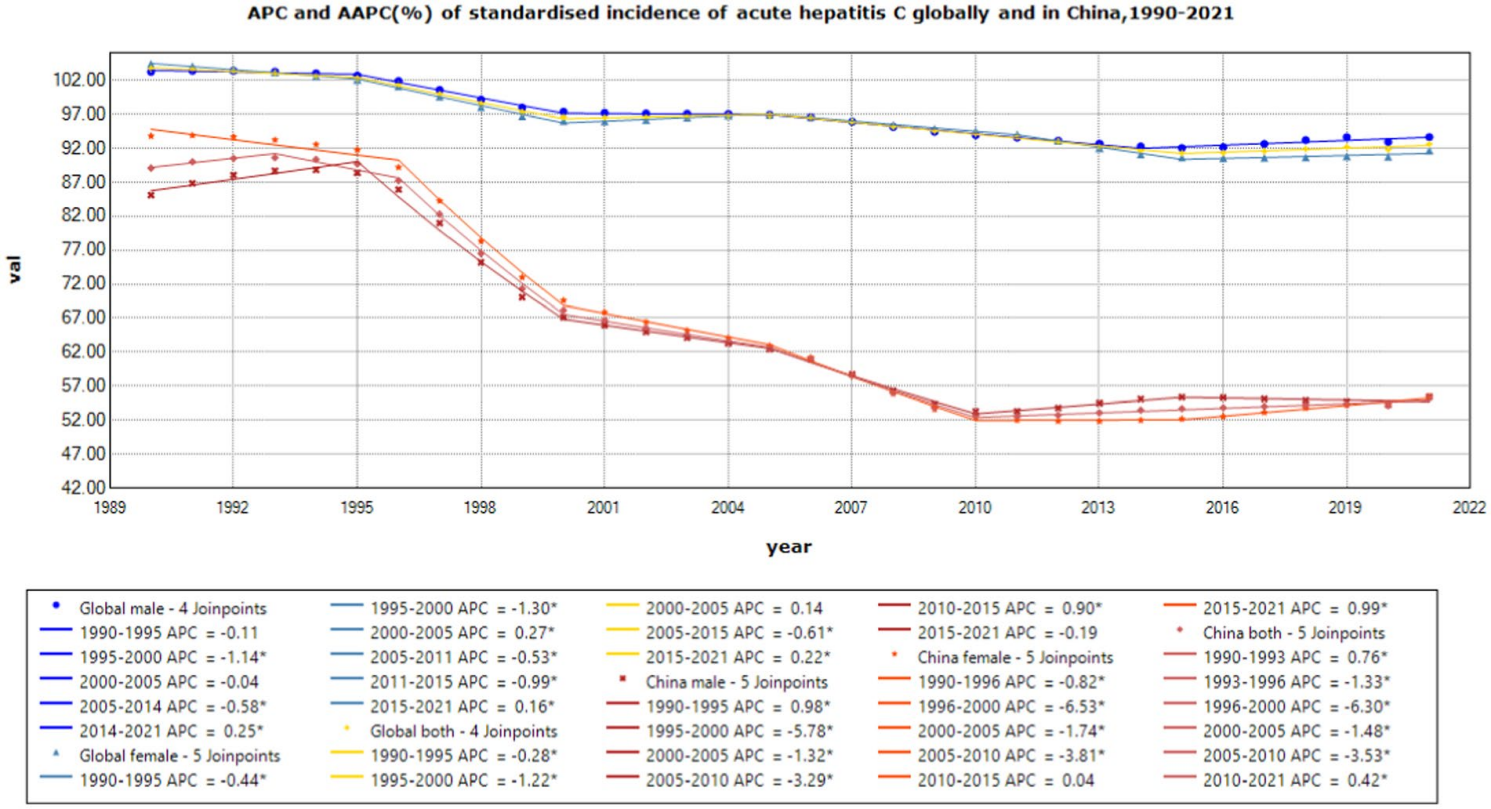
APC and AAPC (%) of standardized incidence of acute hepatitis C globally and in China, 1990–2021.

**Figure 3:**
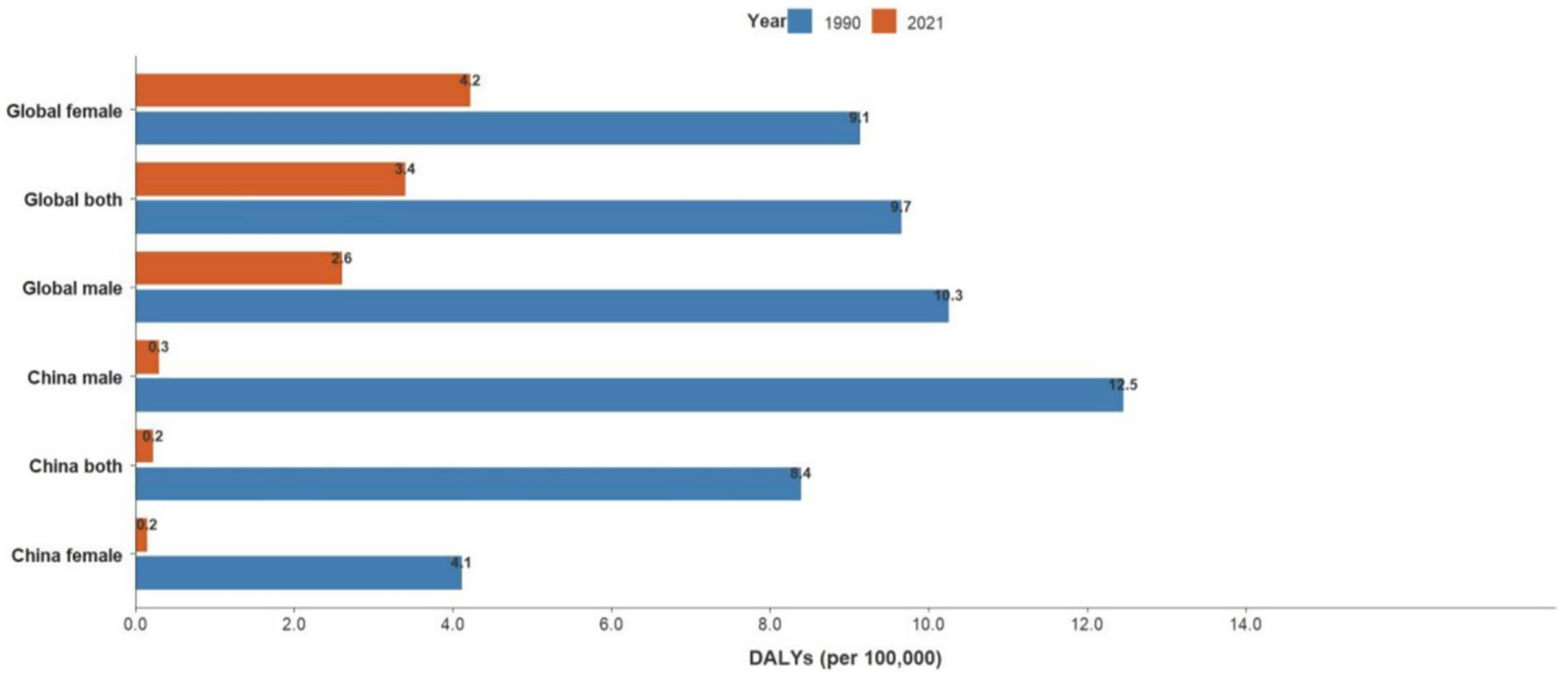
Trends in DALYs for acute hepatitis C: Global vs. China comparison, 1990–2021.

**Figure 4:**
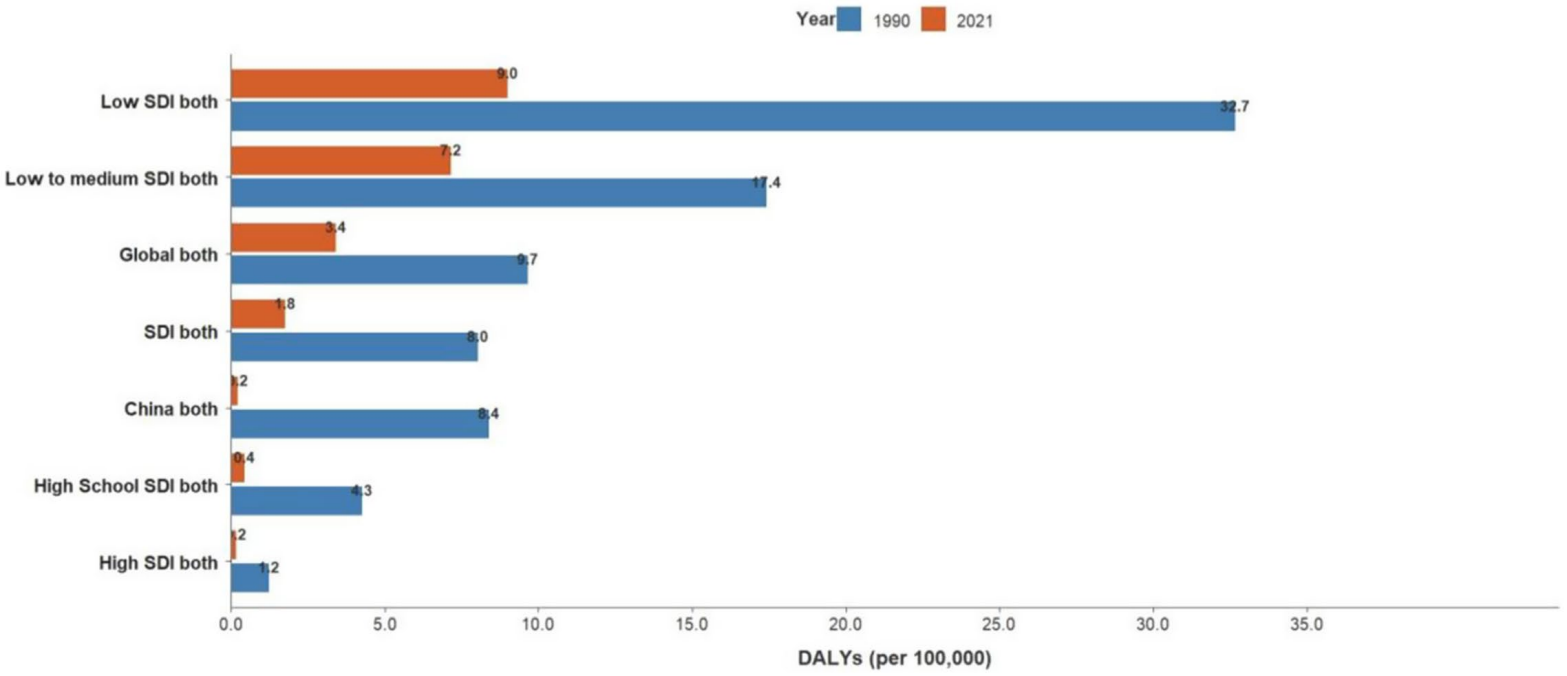
SDI-based disparities in DALYs trends for acute hepatitis C globally, 1990–2021.

**Figure 5:**
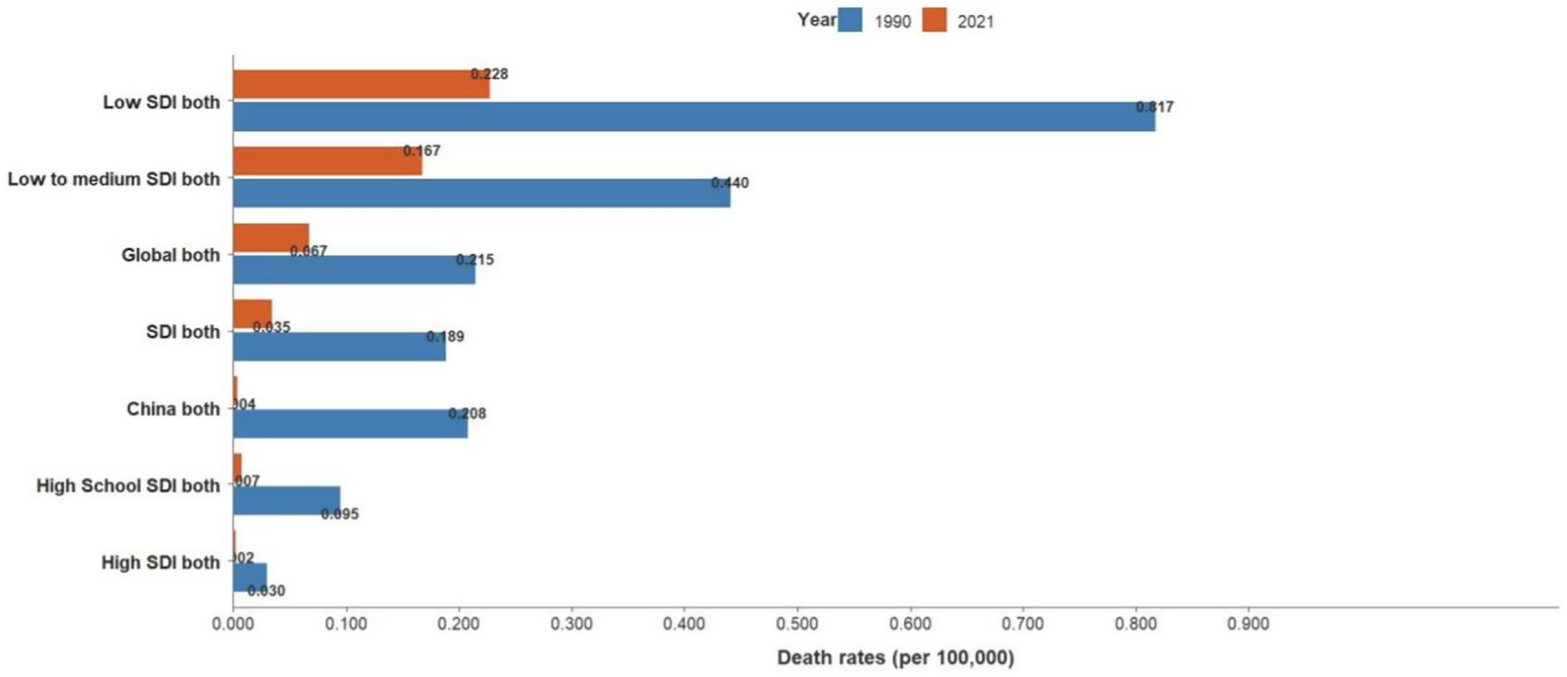
Trends in death rates of acute hepatitis C by SDI group, 1990–2021.

**Figure 6:**
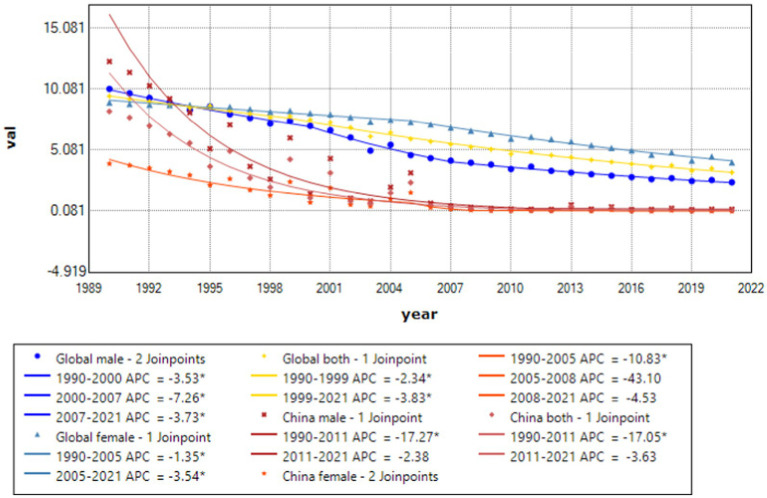
APC and AAPC (%) of YLLs for acute hepatitis C, globally and in China, 1990–2021.

**Figure 7:**
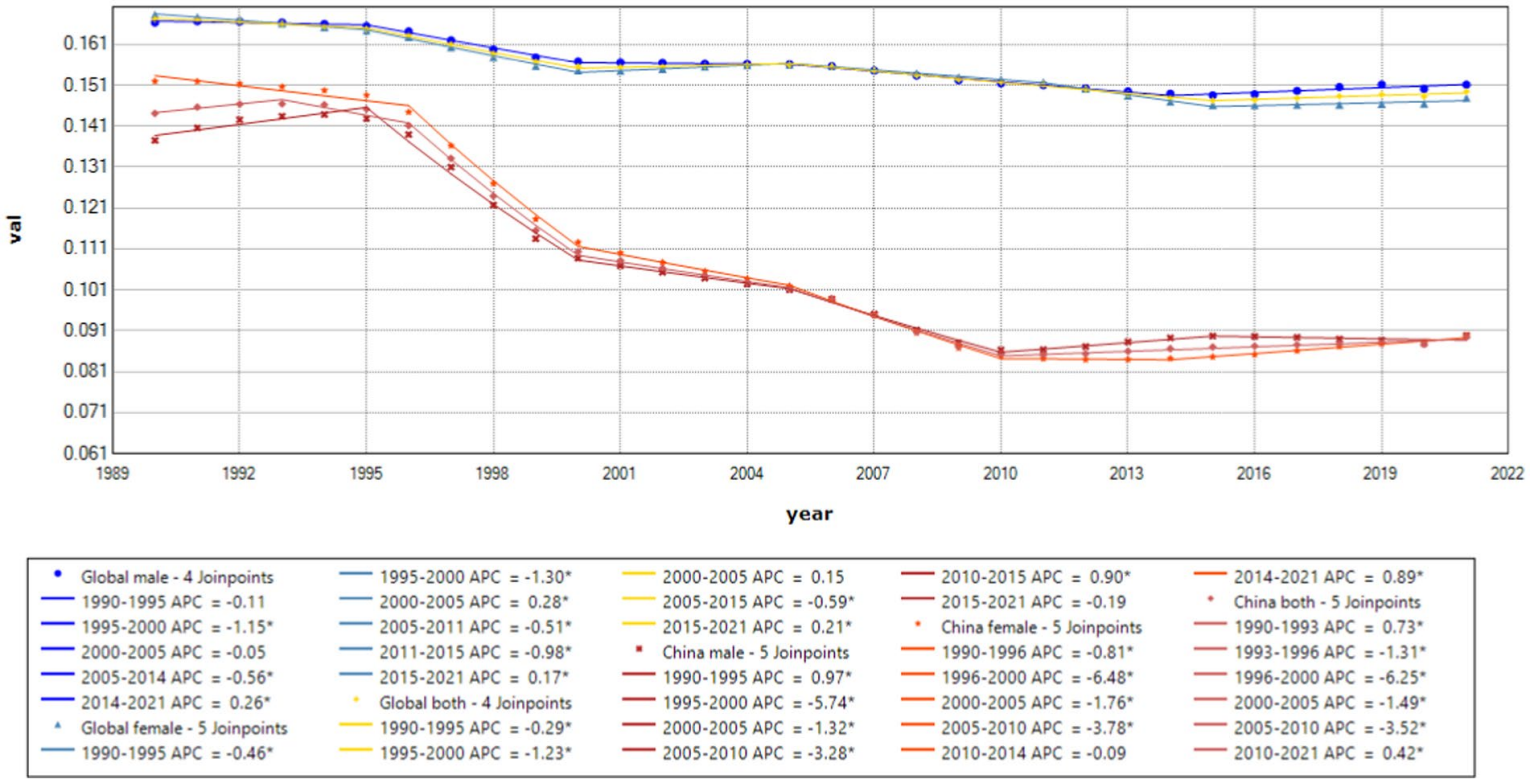
APC and AAPC (%) of YLDs for acute hepatitis C, globally and in China, 1990–2021.

Joinpoint results showed that compared to the global trend, high-middle SDI countries had the fastest decline, averaging 0.85% per year (AAPC = −0.85%, *p* < 0.001), and middle-SDI countries had a faster decline, averaging 0.64% per year (AAPC = −0.64%, *p* < 0.001). It is worth noting that China has the fastest decline in the standardized incidence rate of acute hepatitis C. The standardized incidence rate of acute hepatitis C in China declined by an average of 1.55% per year from 1990 to 2021 (AAPC = −1.55%, *p* < 0.001), from 89.07 per 100,000 in 1990 to 55.22 per 100,000 in 2021. The incidence rate of acute hepatitis C in males declined by an average of 1.42% per year from 1990 to 2021 (AAPC = −1.42%, *p* < 0.001), from 85.1 per 100,000 in 1990 to 55.42 per 100,000 in 2021, and the incidence rate of acute hepatitis C in females declined by an average of 1.7% per year from 1990 to 2021 (AAPC = −1.7%, *p* < 0.001), from 93.81 per 100,000 in 1990 to 55.4 per 100,000 in 2021, and the standardized incidence rate of acute hepatitis C in China started to be lower than the average standardized incidence rate of high-SDI countries in 2008. The global acute hepatitis C death rates all showed a decreasing trend, with the fastest decline from 2001 to 2004, averaging 6.41% per year (APC = −6.41%, *p* < 0.001), of which China had the fastest rate of decline, with an average annual decline of 12.14% from 1990 to 2021 (AAPC = −12.14%, *p* < 0.001), and low-SDI countries remained the countries with the highest death rates. DALYs trend: From 1990 to 2021, global DALYs showed a declining trend over time, with a more pronounced decrease among males. The global DALY rates for males decreased by an average of 4.29% annually, whereas those for females decreased by an average of 2.44% annually. Among the SDI subgroups, the high-middle SDI declined the fastest, by an average of 7.17% per year, and the low-middle SDI declined the slowest, by an average of 2.82% per year. It is worth noting that the high SDI declined rapidly from 2003 to 2007 (APC = −26.11%, *p* < 0.001). A more rapid decline in DALY rates was observed in China compared to the global trend, with an average annual decline of 11.45% for Chinese men and 11.05% for women (see Table 1 and Figures 1–7).

**Table 1:** APC and AAPC (%) of the standardized incidence of acute hepatitis C globally and in China, 1990–2021.

Location	Year	APC	*P*	AAPC (95%CL)	*P*
China	1990–1993	0.76*	0.005	−1.55 (–1.59 to –1.51)	< 0.001
China	1993–1996	−1.33*	< 0.001		
China	1996–2000	−6.30*	< 0.001		
China	2000–2005	−1.48*	0.001		
China	2005–2010	−3.53*	0.004		
China	2010–2021	0.42*	0.002		
Global	1990–1995	−0.28*	< 0.001	−0.38 (–0.39 to –0.36)	< 0.001
Global	1995–2000	−1.22*	< 0.001		
Global	2000–2005	0.14*	0.014		
Global	2005–2015	−0.61*	< 0.001		
Global	2015–2021	0.22*	< 0.001		
High School SDI	1990–1995	−0.05	0.488	−0.85 (–0.86 to –0.82)	< 0.001
High School SDI	1995–2000	−2.84*	0.002		
High School SDI	2000–2005	−0.63*	0.001		
High School SDI	2005–2010	−1.63*	< 0.001		
High School SDI	2010–2015	−0.34*	0.004		
High School SDI	2015–2021	0.23*	< 0.001		
High SDI	1990–1996	−0.15*	0.008	−0.43(−0.45 to −0.42)	< 0.001
High SDI	1996–2000	−0.88*	0.002		
High SDI	2000–2005	−1.88*	< 0.001		
High SDI	2005–2011	−0.20*	0.006		
High SDI	2011–2015	−0.50*	< 0.001		
High SDI	2015–2019	0.94*	< 0.001		
High SDI	2019–2021	−0.02	0.903		
Low SDI	1990–1995	−0.18*	0.001	−0.42(−0.43 to −0.41)	< 0.001
Low SDI	1995–2002	−0.00	0.982		
Low SDI	2002–2006	−0.40*	0.019		
Low SDI	2006–2015	−0.94*	< 0.001		
Low SDI	2015–2019	−0.41*	< 0.001		
Low SDI	2019–2021	−0.12	0.055		
Low to medium SDI	1990–1995	−0.74*	< 0.001	−0.39(−0.40 to −0.37)	< 0.001
Low to medium SDI	1995–2000	−1.24*	< 0.001		
Low to medium SDI	2000–2005	0.74*	< 0.001		
Low to medium SDI	2005–2011	−0.29*	0.002		
Low to medium SDI	2011–2014	−1.24*	< 0.001		
Low to medium SDI	2014–2021	−0.05	0.222		
Medium SDI	1990–1995	−0.59*	< 0.001	−0.64(−0.66 to −0.62)	< 0.001
Medium SDI	1995–2000	−2.30*	< 0.001		
Medium SDI	2000–2005	−0.05	0.422		
Medium SDI	2005–2014	−0.80*	< 0.001		
Medium SDI	2014–2021	0.29*	< 0.001		

* Indicates that the Annual Percent Change (APC) is significantly different from zero at the alpha = 0.05 level.

### 3.2 Decomposition of effects: age-period-cohort analysis

#### 3.2.1 Age effect

**Figure 8:**
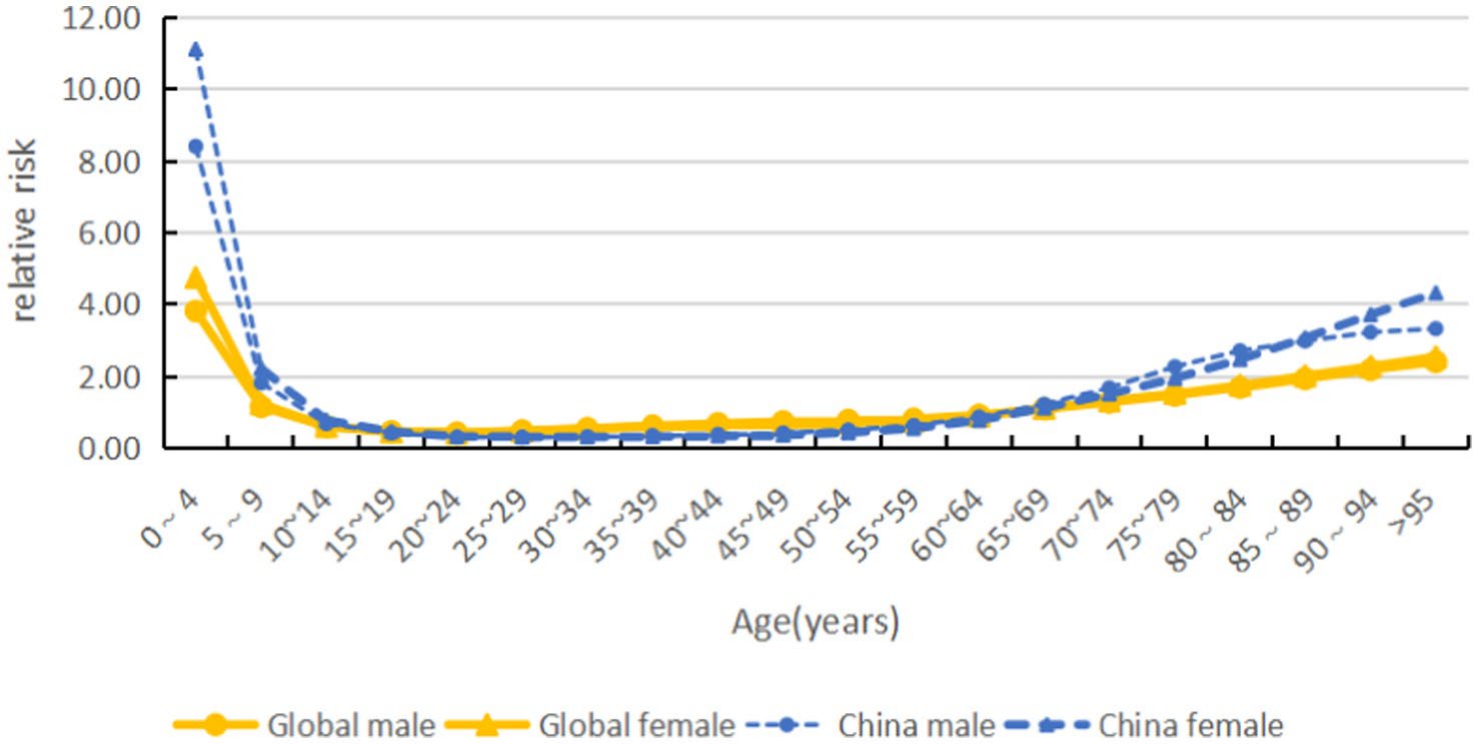
Age effects on acute hepatitis C incidence by sex and country (global and China), 1990–2021.

**Figure 9:**
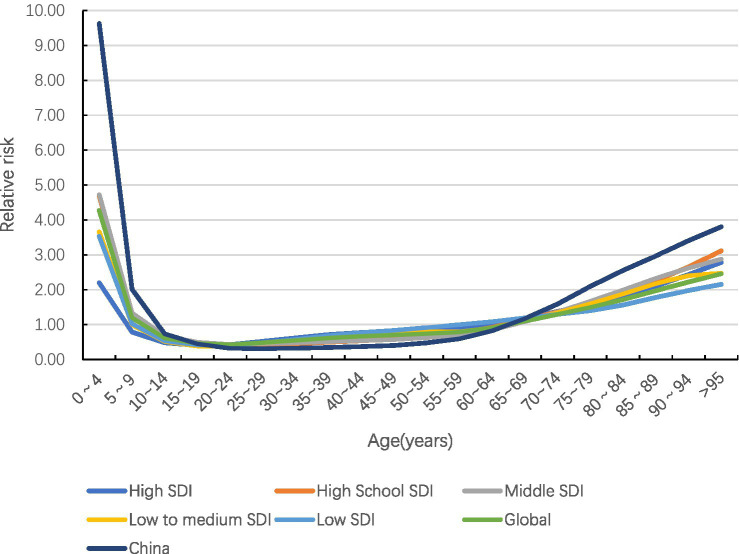
Age-specific effects on acute hepatitis C incidence by SDI group, 1990–2021.

The global incidence risk of acute hepatitis C shows a trend of ‘bimodal’ with increasing age, with the highest incidence risk in the age group of 0–4 years, followed by the age group of 95 years and above, and the trough in the age group of 15–64 years. In the same age group, the risk is higher in women than in men. China’s incidence risk is much higher than that of the world in both younger and older age groups (see Figures 8, 9).

#### 3.2.2 Time effect

**Figure 10:**
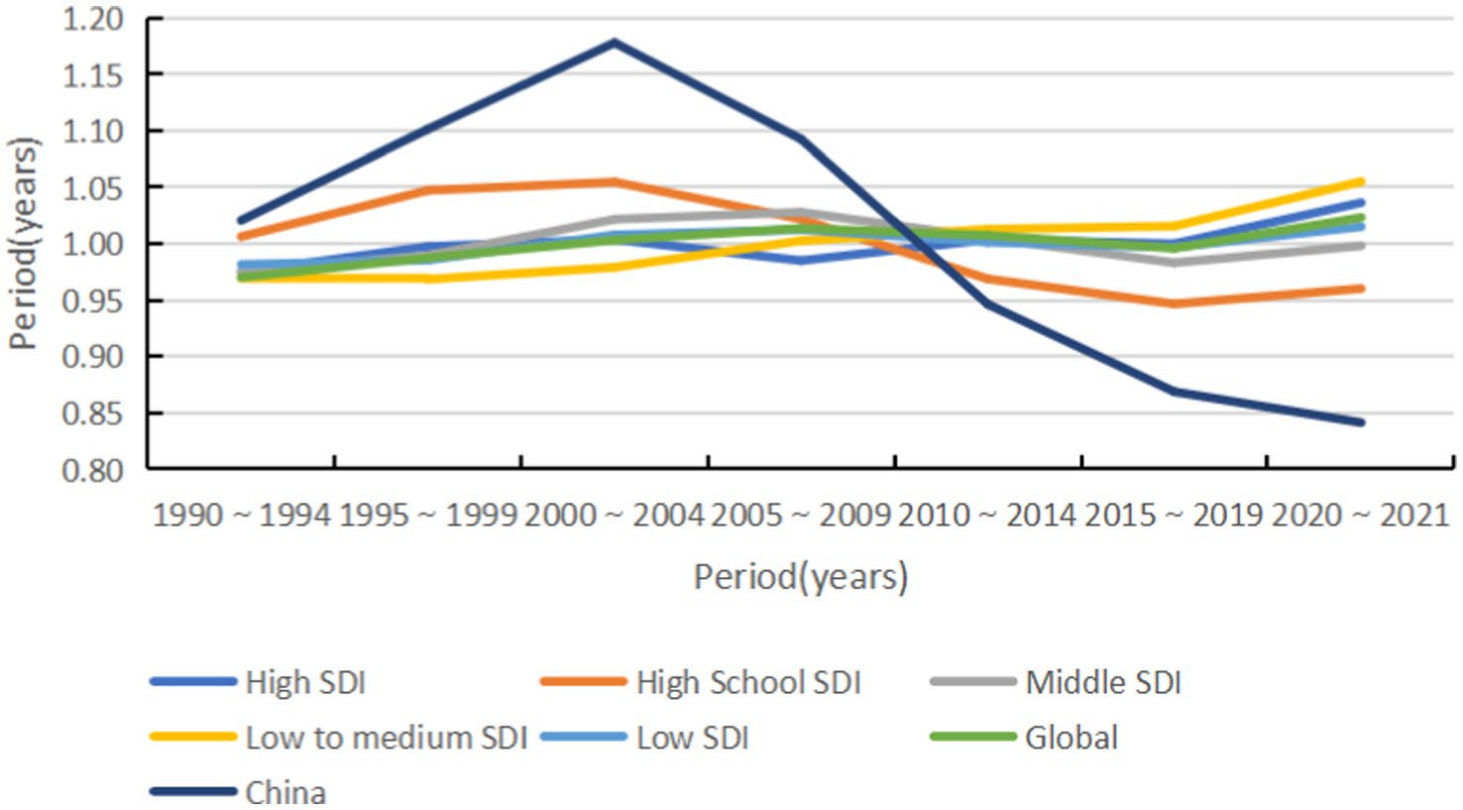
Period effects on acute hepatitis C incidence by SDI group, 1990–2021.

**Figure 11:**
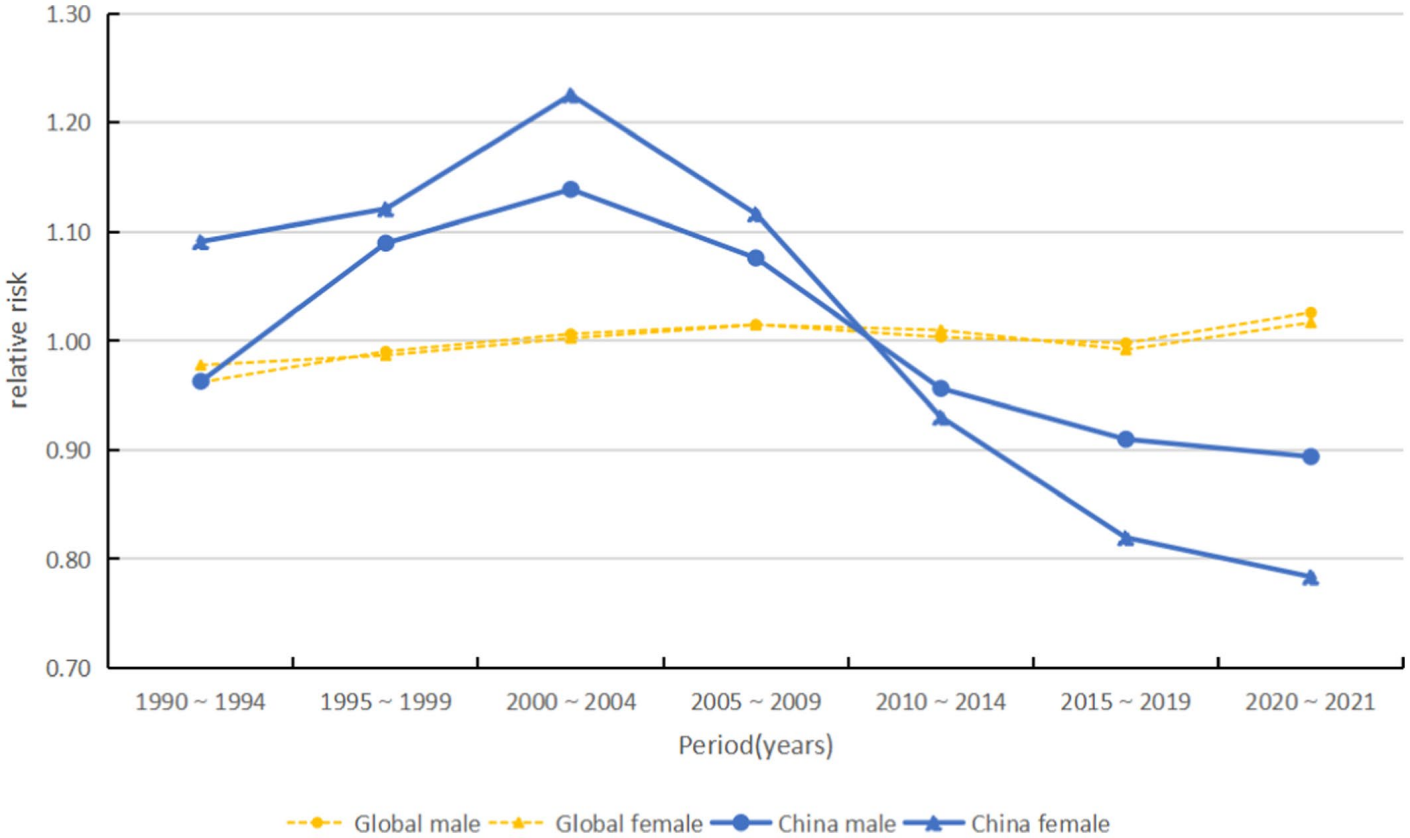
Period effects on acute hepatitis C incidence by sex (global and China), 1990–2021.

From 1990 to 2014, there was no significant trend in the global risk of acute hepatitis C, which began to rise from 2015 to 2019 and was highest from 2020 to 2021. Among them, the incidence risk in high-middle SDI countries gradually decreased over time, while the incidence risk in the remaining subgroups of countries increased. Notably, the risk of acute hepatitis C incidence in China peaked in 2000–2004 and then declined gradually over time at the fastest rate (see Figures 10, 11).

#### 3.2.3 Cohort effect

**Figure 12:**
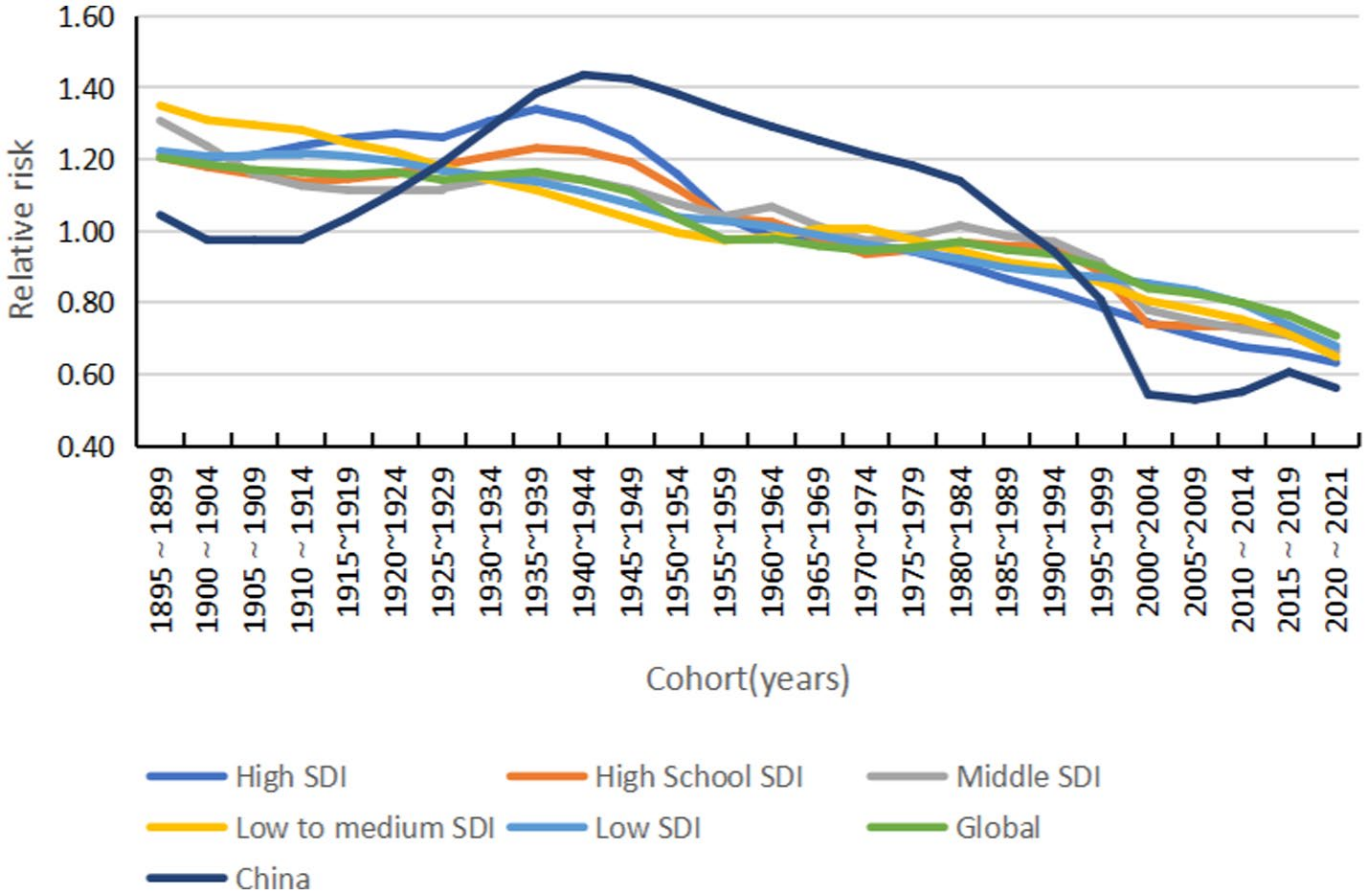
Cohort effects on acute hepatitis C incidence by SDI group, 1990–2021.

**Figure 13:**
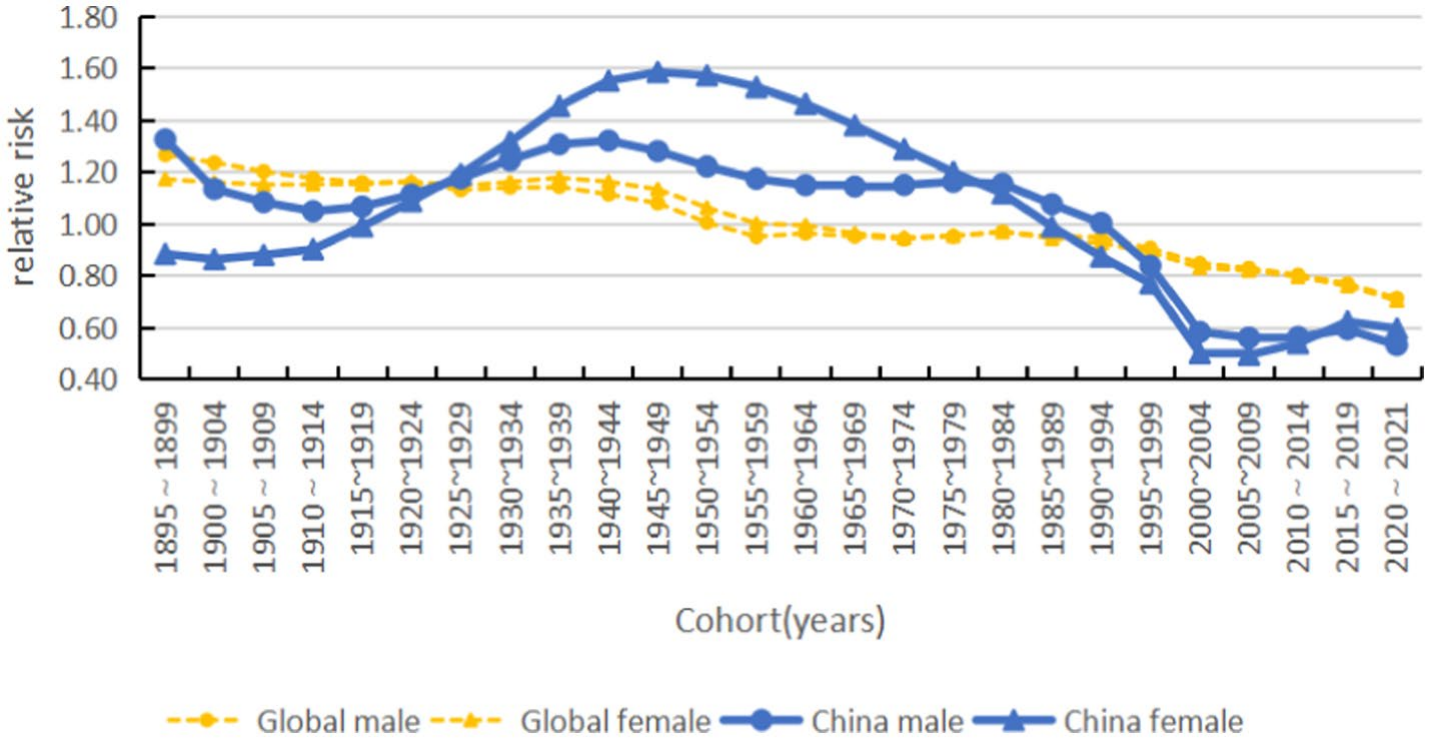
Cohort effects on acute hepatitis C incidence by sex (global and China), 1990–2021.

Morbidity risk changes with the birth cohort in a general downward trend. The 2020–2021 low-SDI cohort has the highest morbidity risk, and high-SDI countries have the lowest morbidity risk. China’s morbidity risk is lower than the average for high-SDI countries, and women born from 2015 to 2021 have a higher morbidity risk than men (see Figures 12, 13).

## 4 Discussion

Based on the data of GBD 2021, this study systematically analysed the long-term trend of acute hepatitis C incidence in the world and China from 1990 to 2021 and revealed the effects of socio-economic development, age structure, time period change, and birth cohort on the incidence risk through the APC model. The results showed substantial differences in incidence rates across SDI categories, with low-SDI countries exhibiting the highest standardized incidence rates of acute hepatitis C, far exceeding the global average. Globally, the standardized incidence of acute hepatitis C shows an overall downward trend. Notably, a particularly rapid decline was observed in China. Women face a higher risk of infection than men within the same age group, and low-SDI countries continue to have the highest death rates. The global risk of acute hepatitis C was “bimodal” with increasing age, with the highest incidence risk in the age group of 0–4 years, followed by the age group of 95 years and above, and the fastest decline in incidence rate in China. The global risk of acute hepatitis C began to rise between 2015 and 2019, peaking in 2020–2021.

In this study, we found that the standardized incidence of acute hepatitis C was significantly higher in low-SDI countries than in high-SDI countries, which is consistent with previous studies (7). The high incidence in low-SDI regions can be attributed to multiple factors: risky healthcare practices, unsafe injections, insufficient screening of blood products, and the reuse of medical devices are the main routes of HCV transmission. The rate of HCV infection due to transfusion safety breaches is as high as 20% in some parts of Africa (8). Low coverage of screening and intervention programmes targeting high-risk groups results in the persistence of hidden chains of transmission (9). The risk of transmission is further exacerbated by poor sterilisation in traditional medical practice (10). In contrast, high-SDI countries have significantly reduced incidence through systematic prevention and control measures. Since the 1990s, Europe and the United States have implemented rigorous blood donor screening and viral inactivation techniques, which have reduced the rate of transfusion-associated HCV infection to <0.001% (11). Since 2014, the widespread use of direct-acting antiviral agents (DAAs), primarily for chronic HCV infection, has achieved cure rates of >95%. By rapidly curing existing chronic infections, DAAs reduce the reservoir of transmissible viruses in the community. This “treatment-as-prevention” effect consequently lowers the incidence of new acute infections and, thereby, the future risk of chronicity at the population level (12). Since 2008, China’s ASIR has remained at a level comparable to or below that of high-SDI countries. It is important to note that the Joinpoint acceleration around 2008 occurred before the widespread availability and reimbursement of DAAs in China, which began primarily after 2017. This initial decline in incidence is likely related to early public health interventions, including enhanced blood safety regulations and stricter screening of blood donors implemented in the early 2000s. However, further evidence is needed to confirm the specific impact of these interventions on HCV incidence trends. The increased accessibility of DAAs in later years further consolidated this declining trend (13). The comprehensive prevention benefits of DAAs are constrained by unequal access, with costs and health system barriers disproportionately affecting high-risk populations and low socioeconomic areas, thereby perpetuating transmission reservoirs. However, uneven regional development has resulted in a higher risk in rural areas of central and western China, suggesting the need to strengthen the capacity of primary care (14). It is important to note that significant regional disparities persist within China, mirroring the health inequities observed across SDI groups globally and underscoring the ongoing challenge of ensuring equitable access to interventions. The APC model revealed a persistent ‘bimodal’ age-specific risk pattern for acute hepatitis C globally, characterised by peaks in early childhood (0–4 years) and very old age (≥95 years), with a pronounced trough during working ages (15–64 years). This pattern is likely driven by distinct aetiologies. The early-childhood peak is predominantly attributable to vertical (mother-to-child) transmission, sustained by uneven implementation of prenatal HCV screening, particularly in low-SDI settings (15, 16). In China, the higher relative risk in younger age groups compared to the global average may signal gaps in universal perinatal screening (17). Conversely, the peak in the older adults reflects cumulative historical exposures from periods of less stringent infection control (e.g., unscreened blood transfusions, unsafe injections) during the mid-to-late twentieth century (18), with age-related immunosenescence potentially increasing the severity of acute episodes (19). The consistency of this bimodal pattern underscores it as a robust epidemiological feature stemming from biological vulnerability and cohort-specific historical risks. The higher prevalence in women than in men in the same age group may be related to differences in the frequency of healthcare contacts and hormone-regulated immune responses (20), which need to be targeted to optimize women’s health services. The age-related double-peak structure identified in this study has been reported in HCV burden studies across multiple countries worldwide, indicating that it is not an isolated data phenomenon. The renewed increase in risk during advanced age more likely reflects historical cohort effects: older adults populations experienced unscreened blood products, repeated injections, or iatrogenic exposures during their youth, with long-term latent effects manifesting predominantly in later life (21). Although GBD estimates for the older adults cohort may exhibit some variability due to smaller case numbers, higher mortality rates, and increased data uncertainty, the consistent emergence of an older adults peak across multinational studies suggests that this phenomenon is unlikely to be primarily driven by model instability. The older adults peak is more likely to be driven by cumulative historical exposure, with model variability playing only a secondary role.

The resurgence in the global ASIR beginning around 2015 likely results from a confluence of factors that vary across the SDI spectrum. In many low- and middle-SDI countries, the observed increase may be partially artefactual, reflecting significant improvements in diagnostic capacity and surveillance systems since the early 2010s, which are uncovering a reservoir of previously undiagnosed prevalent and incident cases (21). Additionally, persistent health system gaps in some settings may contribute to ongoing iatrogenic transmission. In contrast, in high-SDI countries, the increase is largely driven by well-documented shifts in risk factors, most notably the opioid crisis, supported by international evidence from North America and parts of Europe, which has increased injection drug use and sustained person-to-person transmission among younger populations (22). The period effect showing the highest risk in 2020–2021 also necessitates consideration of the impact of the COVID-19 pandemic, which disrupted routine hepatitis prevention and treatment services in many settings, potentially causing delayed diagnoses and a backlog of cases being identified as services resumed (23). Therefore, the post-2015 trend represents a composite picture: improved case ascertainment in some areas, genuine epidemic dynamics driven by behavioural risks in others, and the transient disruptive effect of the pandemic. The cohort effect showed a significant reduction in the risk of morbidity in the late-born cohort, corroborating the protective effect of universal access to DAAs and preventive education for the younger generation (24). However, the persistent decline in the risk of morbidity in high-SDI countries and the increase in the risk in low-SDI countries highlight the long-term challenge of unequal distribution of global health resources. Addressing this requires policies that couple targeted screening for high-risk groups with concerted efforts to ensure equitable access to DAAs across all SDI strata.

Despite a long-term decline, the global increase in acute hepatitis C incidence underscores an ongoing public health challenge. Our findings highlight the urgent need to strengthen prevention and screening strategies, particularly in low-SDI regions, among vulnerable age groups, and to implement targeted screening and prevention programmes for key populations. This includes expanding point-of-care testing and harm-reduction services for people who inject drugs in high-incidence areas and ensuring universal prenatal HCV screening to interrupt vertical transmission. There is an urgent need to accelerate equitable access to DAA treatment. Policies should aim to remove financial and systemic barriers to DAAs, particularly in low- and middle-SDI countries, to rapidly reduce the community viral reservoir and achieve the “treatment as prevention” effect, and for high-risk populations, to achieve the goal of hepatitis C elimination.

## 5 Limitations

First, all data used in this study are sourced from the GBD 2021 database. This database generates temporally and spatially comparable disease burden estimates through complex statistical modelling, bias correction, and smoothing algorithms based on multiple data sources, rather than relying solely on raw national surveillance data. While such modelled estimates help address data gaps and enhance comparability across countries, their inherent smoothing mechanisms may reduce sensitivity to short-term fluctuations or localised anomalies in incidence rates. Second, the modelling nature of GBD estimates may affect the precision of this study’s APC decomposition results, particularly when identifying smaller cohort effects. Due to the model’s inherent smoothing mechanism, subtle cohort fluctuations may be absorbed and merged into broader age or period effects, making them difficult to identify as statistically significant independent cohort variations. Finally, due to limited AHC surveillance and screening capabilities in some countries and regions—particularly in low-SDI countries—actual incidence rates may be underestimated. This limitation may further compromise the accuracy of the GBD model estimates in these areas.

## Data Availability

The data analyzed in this study is subject to the following licenses/restrictions: data in GBD database. Requests to access these datasets should be directed to MW, w17376527246@163.com.
